# Sugar Alcohols as Crosslinking Delay Additives for Fracturing Fluids

**DOI:** 10.3390/gels11060457

**Published:** 2025-06-15

**Authors:** Tariq Almubarak, Mohammed I. Alabdrabalnabi, Abdualilah Albaiz, Mohammed Yami

**Affiliations:** EXPEC Advanced Research Center—Saudi Aramco, Dhahran 31311, Saudi Arabia; mohammed.alabdrabalnabi.1@aramco.com (M.I.A.); abdualilah.baiz@aramco.com (A.A.); mohammed.yami.59@aramco.com (M.Y.)

**Keywords:** hydraulic fracturing, rheology, viscosity control, proppant suspension, oxidizers breaker, sustainability

## Abstract

The development of thermally stable fracturing fluids is essential for the effective stimulation of deep and low-permeability reservoirs. The stabilizing additives used in these fluids typically fall into three categories: crosslinking delay molecules, oxygen scavengers, and pH buffers. However, many conventional additives raise toxicity and environmental concerns, prompting the search for safer alternatives. This study investigates the use of sugar alcohols, commonly used as low-calorie sweeteners, as environmentally responsible additives for high-temperature fracturing fluids. A guar-based fluid system was formulated at a pH of 10 and evaluated using a high-pressure high-temperature (HPHT) rheometer under simulated field pumping conditions at 300 °F for a 90 min period. The viscosity was measured at a shear rate of 100 s^−1^, with intermittent low-shear rates introduced to assess the structural recovery and fluid integrity. The effect of sugar alcohol concentration on crosslinking delay was examined across systems containing varying amounts of a zirconium-based crosslinker ranging from 1 to 4 gpt. The results demonstrated that sugar alcohols effectively delayed crosslinking, allowing for controlled viscosity development and improved stability at elevated temperatures. When optimized at concentrations of 2 ppt of the sugar alcohol with 4 gpt of the crosslinker, the fluid generated a peak viscosity of 600 cP after 2.5 min and maintained a viscosity above 300 cP throughout the 90 min test. Breaker results showed a controlled viscosity reduction, with final viscosity values reaching 10 cP. The proppant settling experiments confirmed the suspension of more than 95% of the proppant during the treatment window. These findings highlight the potential of sugar alcohols as effective and environmentally safer crosslinking delay additives for hydraulic fracturing applications.

## 1. Introduction

As deeper, hotter, and tighter formations are targeted, the demand for high-temperature hydraulic fracturing fluids continues to increase. These environments require fluids capable of retaining viscosity and elasticity under elevated thermal and mechanical stresses.

An ideal fracturing fluid uses polymers that hydrate effortlessly in water (<5 min), withstand the mechanical shearing of high-pressure pumps on the surface, crosslink, and generate high viscosity as it reaches 80% of the target zone depth. A typical timeframe for this property depends on the depth of the target zone and can range between 2 and 5 min. The fluid is pumped at pressures higher than the fracture pressure to generate fractures in the target zone. The aim of the hydraulic fracturing fluid is to generate sufficient rheological properties to transport the proppant into the generated fractures. Maintaining a high viscosity is challenging, especially under continuous shearing at high-temperature conditions. The final step in a hydraulic fracturing treatment is to break the crosslinked polymer network to lower the fluid viscosity and to easily recover it, preventing formation damage.

**Figure 1:**
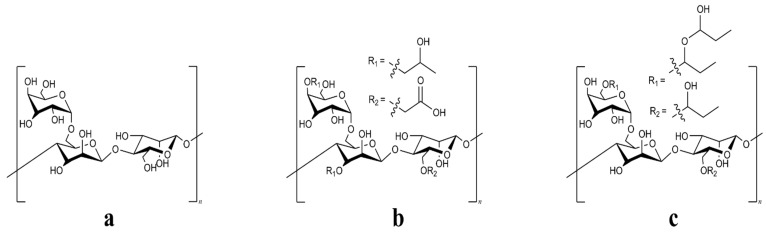
Structural formula of (**a**) guar gum and its derivatives (**b**) CMHPG and (**c**) HPG.

Guar derivatives, as seen in Figure 1, are known natural polysaccharides used in fracturing fluids to provide viscosity and are typically crosslinked with metallic crosslinkers such as zirconium to enhance proppant transport [1,2,3]. Compared to guar and hydroxypropyl guar (HPG), carboxymethylhydroxypropyl guar (CMHPG) dissolves more easily in water and achieves higher thermal stability. This is because its structure includes both carboxymethyl and hydroxypropyl groups, which improve hydration and help the polymer expand in solution due to charge repulsion [4]. Nonetheless, at elevated temperatures, fluids prepared by any of these mentioned polymers are prone to degradation, leading to rapid viscosity loss [5].

To mitigate this, stabilizing additives are employed to improve thermal performance. These fall into three main categories: crosslinking delay molecules, oxygen scavengers, and pH buffers [6]. Among them, compounds such as sodium sulfite and sodium thiosulfate are widely used but have drawbacks. Sodium sulfite can promote sulfate-reducing bacterial growth, while sodium thiosulfate may thermally decompose to produce hydrogen sulfide (H_2_S), introducing corrosion and safety risks in the oilfield [7,8].

Crosslinking delay additives offer a more direct method of controlling gelation timing, enhancing pumpability and thermal resilience. Common delay agents include triethanolamine, propylene glycol, alpha-hydroxy acids, and bicarbonates [9,10,11]. However, many such agents are associated with toxicity, environmental persistence, or operational inefficiencies. For example, triethanolamine is known for its strong odor and carcinogenicity with prolonged exposure [12,13,14]. Reviews of hydraulic fracturing fluids have identified hundreds of chemical constituents, many of which are flagged as harmful to human health or aquatic ecosystems, prompting regulatory scrutiny [15,16].

These challenges underscore the need for additives that are both operationally effective and environmentally benign. Ideally, such additives would delay crosslinking, enhance fluid stability at high temperatures, and integrate seamlessly into existing formulations without introducing toxicity or handling risks. The literature increasingly supports the use of plant-derived and biodegradable compounds, which show low mammalian and aquatic toxicity, environmental degradability, and minimal interference with soil microbiota [17,18,19].

In parallel, industry-wide efforts are shifting toward sustainable practices, such as recycling chemical additives and using alternative water sources like produced water and seawater, reducing environmental impact and freshwater consumption [20,21,22,23,24,25]. The development of safer fluid chemistries, such as sugar-based additives, aligns closely with these goals.

Sugar and its derivatives serve functional roles in industrial processes due to their versatile chemical properties. These carbohydrates, comprising carbon, hydrogen, and oxygen, are classified into monosaccharides (e.g., glucose and fructose), disaccharides (e.g., sucrose), and polysaccharides (e.g., starch, guar, cellulose). Polysaccharides, particularly guar-based materials, are widely utilized in oil and gas operations as viscosifying agents in hydraulic fracturing fluids [26,27].

Sucrose, the most consumed disaccharide, consists of glucose and fructose linked by an α,β-glycosidic bond. Industrial refining from sugarcane and sugar beet yields high-purity sugar and molasses, the latter being rich in simple sugars and trace minerals [28,29,30,31,32,33,34]. Sugar plays an essential role in human metabolism [35]. However, excessive consumption is linked to obesity and a variety of disorders [36,37,38,39,40]. Consequently, natural and artificial sugar substitutes were introduced [41,42,43,44]. While these sugars are not used directly in fracturing fluids, the structural and functional insights from sugar chemistry inform the development of sugar-derived additives for oilfield applications.

**Figure 2:**
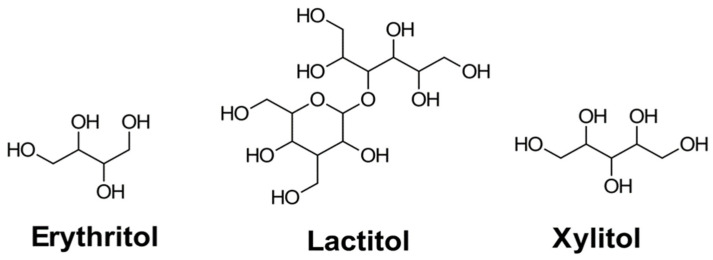
Structural formula (without considering stereochemistry) of common sugar alcohols, including erythritol, xylitol (polyols), and lactitol (disaccharide alcohol), used as low-calorie sweeteners.

To enhance safety and sustainability, research has turned bio-derived alternatives to conventional fluid additives. Natural and synthetic sugar substitutes such as sugar alcohols (polyols) have emerged as promising candidates. These include compounds such as erythritol, lactitol, and xylitol, which possess hydroxyl-rich, non-reducing structures that improve solubility, stability, and compatibility with aqueous systems [45,46,47,48]. Sugar alcohols occur naturally in fruits or are synthesized from sugars or starches, as seen in Figure 2.

Their broad use in pharmaceutical and food products highlights their low toxicity and favorable safety profile [49,50,51,52]. Their hydroxyl-rich structures are chemically analogous to the side chains of guar polymers. Their ability to form hydrogen bonds and their structural similarity to guar suggest potential for interactions with crosslinking agents, enabling delayed gelation and improved thermal control in fracturing fluids.

Although polyols have been previously used in formulations for hydration control and moisture retention, their systematic evaluation as crosslinking delay additives in high-temperature fracturing fluids remains absent from the literature.

This study addresses that gap by investigating the performance of sugar alcohols in zirconium-crosslinked CMHPG fluids. The work evaluates their influence on gelation kinetics, viscosity stability at 300 °F, compatibility with oxidative breakers, and proppant suspension. These findings aim to support the development of cleaner, more controllable fracturing fluid systems for high-temperature field operations.

## 2. Results and Discussion

### 2.1. Baseline Crosslinked Viscosity

**Figure 3:**
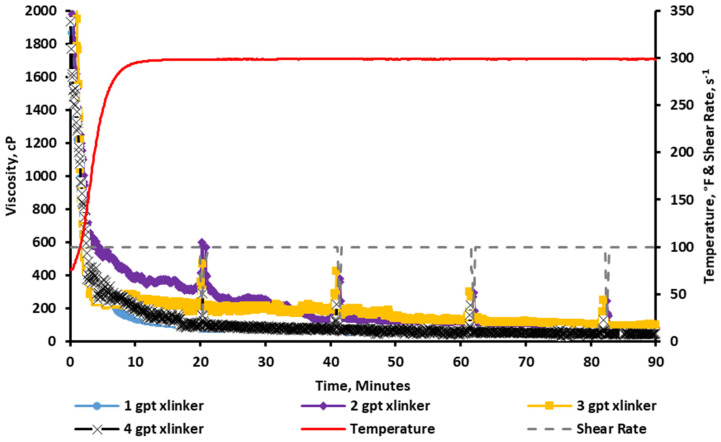
Viscosity profile of CMHPG fracturing fluid crosslinked with 1–4 gpt of Zr-lactate at a pH of 10.7 and 300 °F. Fluids exhibited rapid crosslinking and poor shear stability in the absence of sugar alcohol.

The fracturing fluids are tested without the addition of the sugar alcohol at crosslinker concentrations of 1, 2, 3, and 4 gpt, a pH of ~10.7, and 300 °F to generate a baseline value to be used as a “control” experiment for comparison with upcoming tests with the additives. The results show that the viscosity value for all crosslinker concentrations did not generate significant rheological stability under these conditions, as seen in Figure 3. This was due to the instant crosslinking that occurred as soon as the crosslinker was added to the fluid at these pH conditions, which generated a significantly high initial viscosity that dropped to a low value (below 100 cP) as the fluid was sheared. Additionally, this viscosity breakdown did not exhibit a re-healing property. Although all these tests resulted in failure, the figure shows that the viscosity profile was able to hold a relatively higher viscosity at a concentration of 2 and 3 gpt of the crosslinker.

### 2.2. Tests with Varying Crosslinker and Sugar Alcohol Concentrations

Each crosslinker concentration was subsequently tested with varying amounts of sugar alcohols to study its influence on the rheological performance.

#### 2.2.1. Rheology at a 1 gpt Crosslinker Concentration

**Figure 4:**
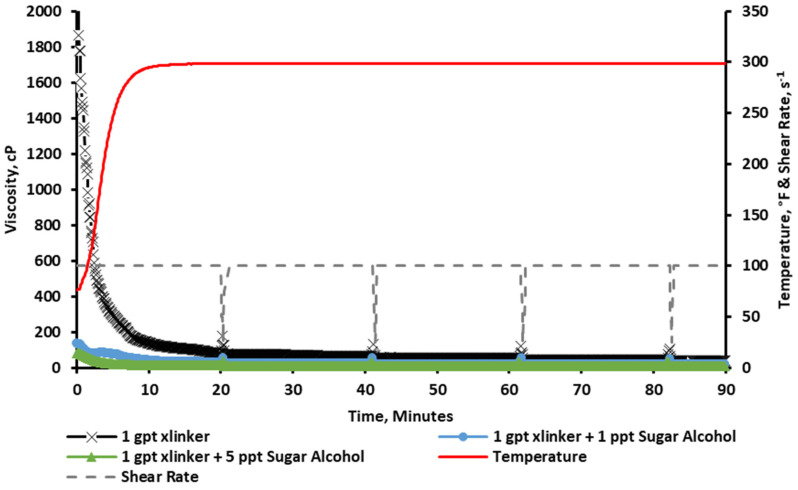
Viscosity of CMHPG fluid crosslinked with 1 gpt of Zr-lactate and 0, 1, and 5 ppt of sugar alcohol at a pH of 10.7 and 300 °F. Addition of sugar alcohol reduced viscosity below acceptable thresholds, suppressing effective gelation.

The results in Figure 4 show that the baseline viscosity resulted in an instant crosslinking (vortex closure ~ 1 s), and the viscosity was reduced significantly throughout the test. Following that, sugar alcohol concentrations of 1 and 5 ppt were added to the fracturing fluid and crosslinked at 1 gpt of Zr-lactate crosslinker for comparison. The results show that the corresponding amount of sugar alcohol significantly reduced viscosity, rendering it below the acceptable limits (below 100 cP for the pumping duration).

#### 2.2.2. Visual Observation at a 1 gpt Crosslinker Concentration

**Figure 5:**
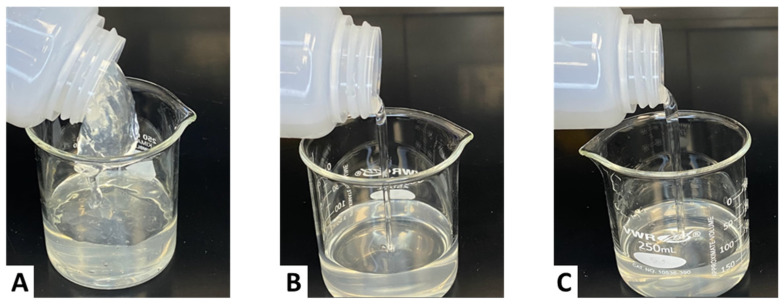
Lipping behavior of CMHPG fluid crosslinked with 1 gpt of Zr-lactate at a pH of 10.7 and 300 °F: (**A**) 0 ppt of sugar alcohol—thick gel, no re-healing; (**B**) 1 ppt of sugar alcohol—weak, water-like flow; (**C**) 5 ppt of sugar alcohol—minimal viscosity, water-like flow.

Figure 5 illustrates the lipping behavior of CMHPG fluids crosslinked with 1 gpt of zirconium lactate and varying sugar alcohol concentrations. The base case without sugar alcohol (Case A) exhibited a thick, crosslinked structure capable of forming a lip when tilted. However, after shearing, the fluid did not recover, indicating a brittle, non-healing gel structure. This behavior aligns with the viscosity drop seen in Figure 4, confirming a lack of thermal resilience.

Cases B and C, containing 1 and 5 ppt of sugar alcohol, respectively, both displayed water-like flow characteristics. The fluids failed to form a lip and had little to no structure at room temperature, demonstrating that excessive sugar alcohol concentrations suppressed crosslinking entirely. These visual results support the rheological data, showing that over-dosing sugar alcohol leads to underdeveloped viscosity and ineffective proppant transport capability.

#### 2.2.3. Rheology at a 2 gpt Crosslinker Concentration

**Figure 6:**
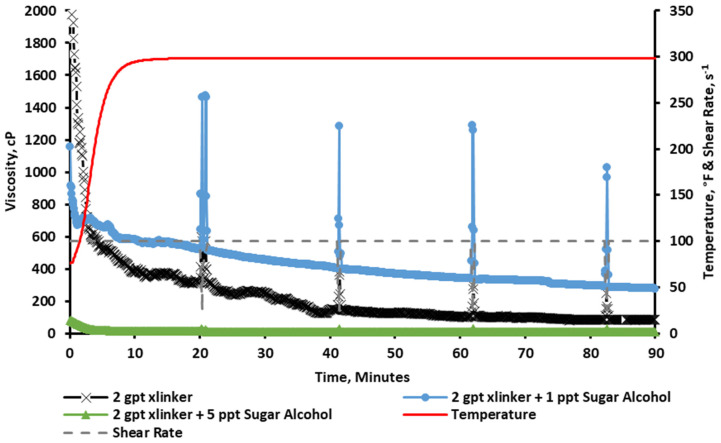
Viscosity of CMHPG fluid crosslinked with 2 gpt of Zr-lactate and 0, 1, and 5 ppt of sugar alcohol at a pH of 10.7 and 300 °F. A 1 ppt concentration delayed crosslinking and improved stability; higher concentrations weakened viscosity.

The results in Figure 6 show that the baseline viscosity resulted in an instant crosslinking (vortex closure ~ 1 s), and it was easily broken as the fluid was sheared through the test, reducing its viscosity. Following that, a sugar alcohol concentration of 1 and 5 ppt was added to the fracturing fluid for comparison. The results show that a concentration of 1 ppt of sugar alcohol was able to improve the rheological stability by slowing down the crosslinking profile (vortex closure ~ 3 s). This resulted in a stable profile generating a viscosity above 300 cP for the duration of pumping. The figure also shows that adding 5 ppt of the sugar alcohol significantly reduced viscosity below the acceptable limits (below 100 cP).

#### 2.2.4. Visual Observation at a 2 gpt Crosslinker Concentration

**Figure 7:**
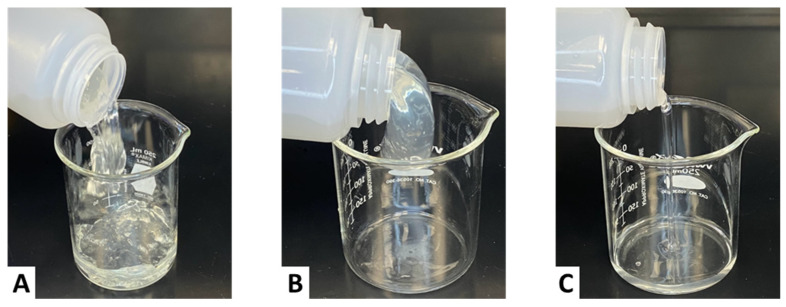
Lipping behavior of CMHPG fluid crosslinked with 2 gpt of Zr-lactate at a pH of 10.7 and 300 °F: (**A**) 0 ppt of sugar alcohol—over-crosslinked, fragmented gel; (**B**) 1 ppt of sugar alcohol—cohesive structure, stable lip; (**C**) 5 ppt of sugar alcohol—water-like flow.

As seen in Figure 7, the fluid without sugar alcohol (Case A) crosslinked too quickly and lacked stability. With 1 ppt of sugar alcohol (Case B), the fluid achieved a stable, uniform texture with clean lipping behavior. Higher concentrations (Case C) broke down viscosity entirely, behaving more like water.

#### 2.2.5. Rheology at a 3 gpt Crosslinker Concentration

**Figure 8:**
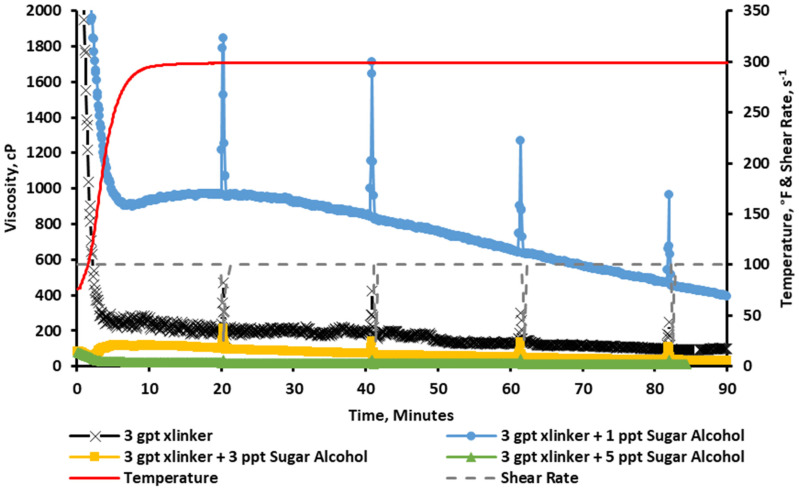
Viscosity of CMHPG fluid crosslinked with 3 gpt of Zr-lactate and 0, 1, 3, and 5 ppt of sugar alcohol at a pH of 10.7 and 300 °F. Optimal stability observed at 1 ppt; 3 ppt exhibited delayed thickening; 5 ppt suppressed viscosity.

The trend in Figure 8 shows that the baseline viscosity resulted in an instant crosslinking (vortex closure ~ 1 s), and it was easily broken as the fluid was sheared through the test, reducing its viscosity. After that, sugar alcohol concentrations of 1, 3, and 5 ppt were added to the fracturing fluid. The results show that a concentration of 1 ppt of sugar alcohol was able to improve the rheological stability by slowing down the crosslinking profile (vortex closure ~ 5 s). The figure also shows that the incorporation of 3 ppt of the sugar alcohol produced an interesting rheological profile. The viscosity was initially low, but as the temperature increased, it started to build its viscosity (this profile is more favorable as it reduces the pumping requirement on the surface by delaying the viscosity buildup). For the last run that incorporated 5 ppt of the sugar alcohol, it was noted that the additive significantly reduced the viscosity below the acceptable limits (below 100 cP).

#### 2.2.6. Visual Observation at a 3 gpt Crosslinker Concentration

**Figure 9:**
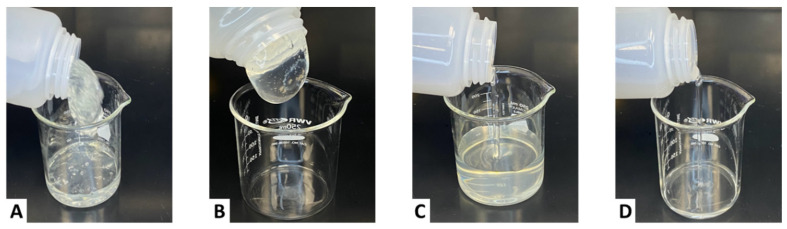
Lipping behavior of CMHPG fluid crosslinked with 3 gpt of Zr-lactate at a pH of 10.7 and 300 °F: (**A**) 0 ppt of sugar alcohol—thick but unstable gel; (**B**) 1 ppt of sugar alcohol—improved cohesion, stable lip; (**C**) 3 ppt of sugar alcohol—low viscosity, water-like flow; (**D**) 5 ppt of sugar alcohol— water-like flow.

As shown in Figure 9, the fluid without sugar alcohol (Case A) formed a thick gel but exhibited a segmented, unstable texture, confirming over-crosslinking. With 1 ppt of sugar alcohol (Case B), the fluid appeared more cohesive and formed a stable lip, suggesting improved gel quality. At 3 and 5 ppt (Cases C and D), the fluids lost structural integrity, flowed freely, and showed no signs of lipping—consistent with suppressed crosslinking and the reduced viscosity profiles seen in Figure 8.

#### 2.2.7. Rheology at a 4 gpt Crosslinker Concentration

**Figure 10:**
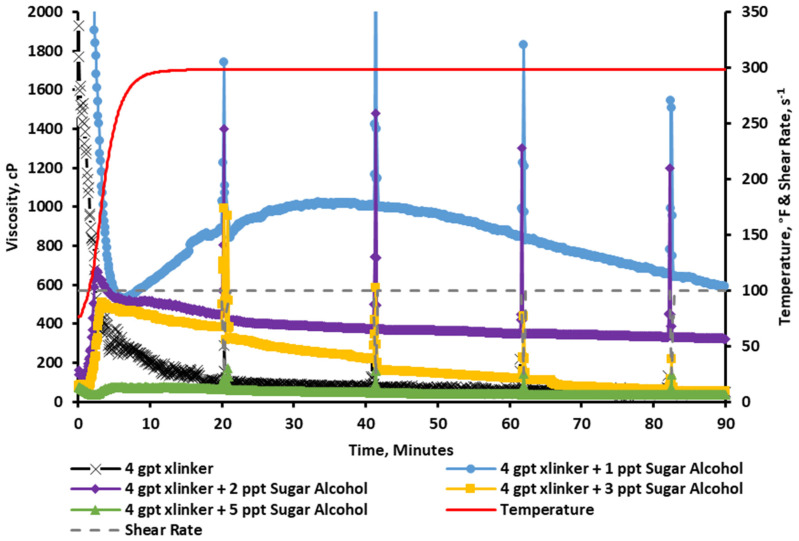
Viscosity of CMHPG fluid crosslinked with 4 gpt of Zr-lactate and 0, 1, 2, 3, and 5 ppt of sugar alcohol at a pH of 10.7 and 300 °F. Sugar alcohol at 2 ppt showed delayed but strong viscosity buildup, outperforming other concentrations.

The results in Figure 10 show that the baseline viscosity resulted in an instant crosslinking (vortex closure ~ 1 s), and the viscosity was reduced as the fluid was sheared through the test. Following that, sugar alcohol concentrations of 1, 2, 3, and 5 ppt were added to the fracturing fluid for comparison. The results show that a concentration of 1 ppt of sugar alcohol was able to improve the rheological stability by slowing down the crosslinking profile (vortex closure ~ 5 s). The key significance here is that the crosslinker was able to build up further viscosity as the test progressed, indicating that the sugar alcohol may act as a crosslinking delay and not just a crosslinking suppressant. The figure also shows that the incorporation of 2 or 3 ppt of the sugar alcohol produced a surprising rheological profile. The viscosity was initially low, but as the temperature increased, it started to build a higher viscosity. In the 2 ppt case, it was able to reach stable viscosity readings of above 300 cP for the duration of pumping. For the last run that incorporated 5 ppt of the sugar alcohol, it was noted that the additive significantly reduced the viscosity below the acceptable limits.

**Figure 11:**
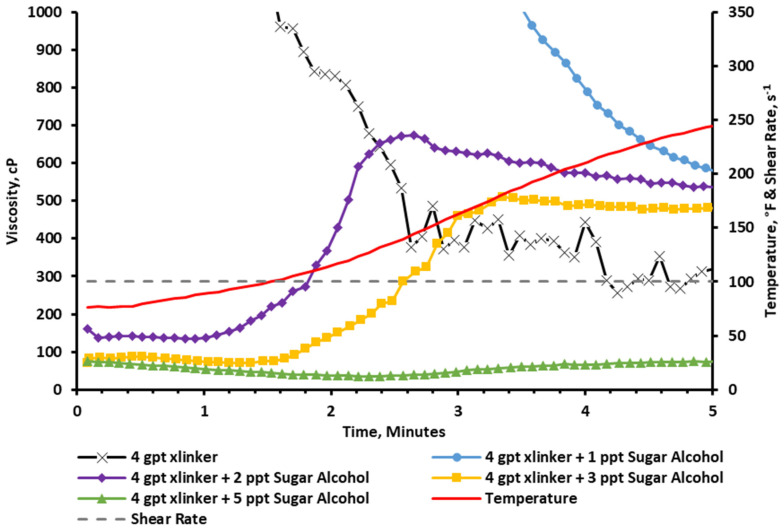
Zoomed-in viscosity profiles during the first 5 min of testing (Figure 10). Fluids with 1–3 ppt of sugar alcohol showed reduced initial viscosity followed by controlled buildup, enabling easier pumping and delayed crosslinking.

A zoomed-in portion of the graph is separated and can be seen in Figure 11. This graph clearly shows that the viscosity profile starts low to ease the pumping operation by reducing frictional pressures, and it increases in performance as the temperature rises, reaching a peak viscosity above 600 cP at around 150 °F and 2.5 min. This type of behavior is highly desired for deep, high-temperature hydraulic fracturing applications.

#### 2.2.8. Visual Observation at a 4 gpt Crosslinker Concentration

**Figure 12:**
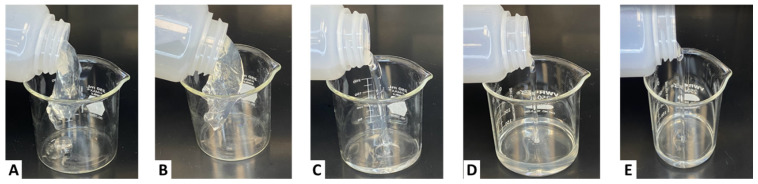
Lipping behavior of CMHPG fluid crosslinked with 4 gpt of Zr-lactate at a pH of 10.7 and 300 °F: (**A**) 0 ppt of sugar alcohol—fragmented, unstable crosslink; (**B**) 1 ppt of sugar alcohol—improved lip, partial re-healing; (**C**) 2 ppt of sugar alcohol—optimal viscosity and structure, water-like flow; (**D**) 3 ppt of sugar alcohol—reduced viscosity, water-like flow; (**E**) 5 ppt of sugar alcohol—no structure, water-like flow.

The fracturing fluid lipping test results are seen in Figure 12. Case A exhibited a viscous behavior that was able to lip. However, Case A showed indications of an unstable crosslinking where the fluid was broken up into separate chunks. Case B exhibited a more full and stable crosslinking behavior that was able to form a lip perfectly. When both fluids were sheared, Case A did not exhibit any re-healing properties, whereas Case B exhibited lipping after shearing. This is confirmed by Figure 10, where the fluid indicated the ability to release the crosslinker over time. Moreover, Cases C, D, and E depicted a water-like behavior where the viscosity was below 200 cP for Case C and below 100 cP for Cases D and E. From these tests, it was decided that the fluid performed best in a combination of 2 ppt of sugar alcohol and 4 gpt of the crosslinker (Case C). Consequently, the breaker and proppant suspension tests were made with this fluid.

### 2.3. Breaker Tests

**Figure 13:**
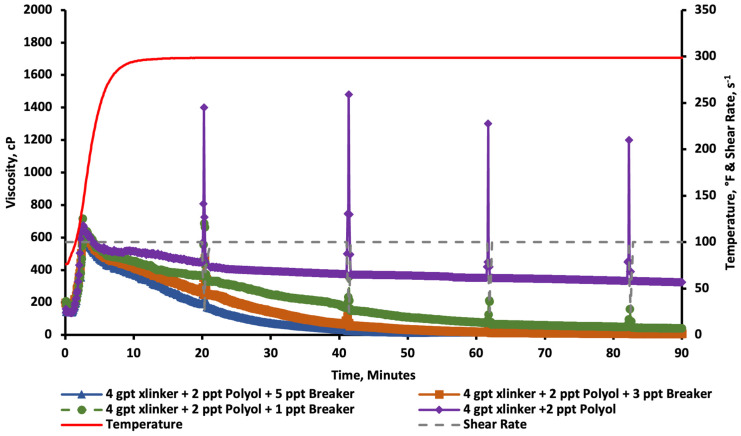
Breaker performance of CMHPG fluid with 4 gpt of Zr-lactate, 2 ppt of sugar alcohol, and varying concentrations (1–5 ppt) of sodium bromate at 300 °F. Viscosity break was controlled and consistent across breaker levels.

An important part of any fracturing fluid is the ability to break it at the end of the treatment to reduce damage to the proppant pack, fracture face, and the formation [53]. Based on this, multiple tests were conducted with sodium bromate as the breaker. The results in Figure 13 show that the fracturing fluid can lose viscosity in the presence of a sodium bromate breaker. The break was controlled, and the viscosity reached values of ~10 cP at the end of the 1.5 h test. The time to lose viscosity was consistent with the increase in breaker concentrations. This test proves the ability to tailor the breaking profile according to the planned pumping duration. Additionally, lowering the viscosity from ~600 to ~10 cP brings comfort to the operators as they plan to flow the fracturing fluid residue and produce hydrocarbons from these generated fractures after the treatment is completed.

**Figure 14:**
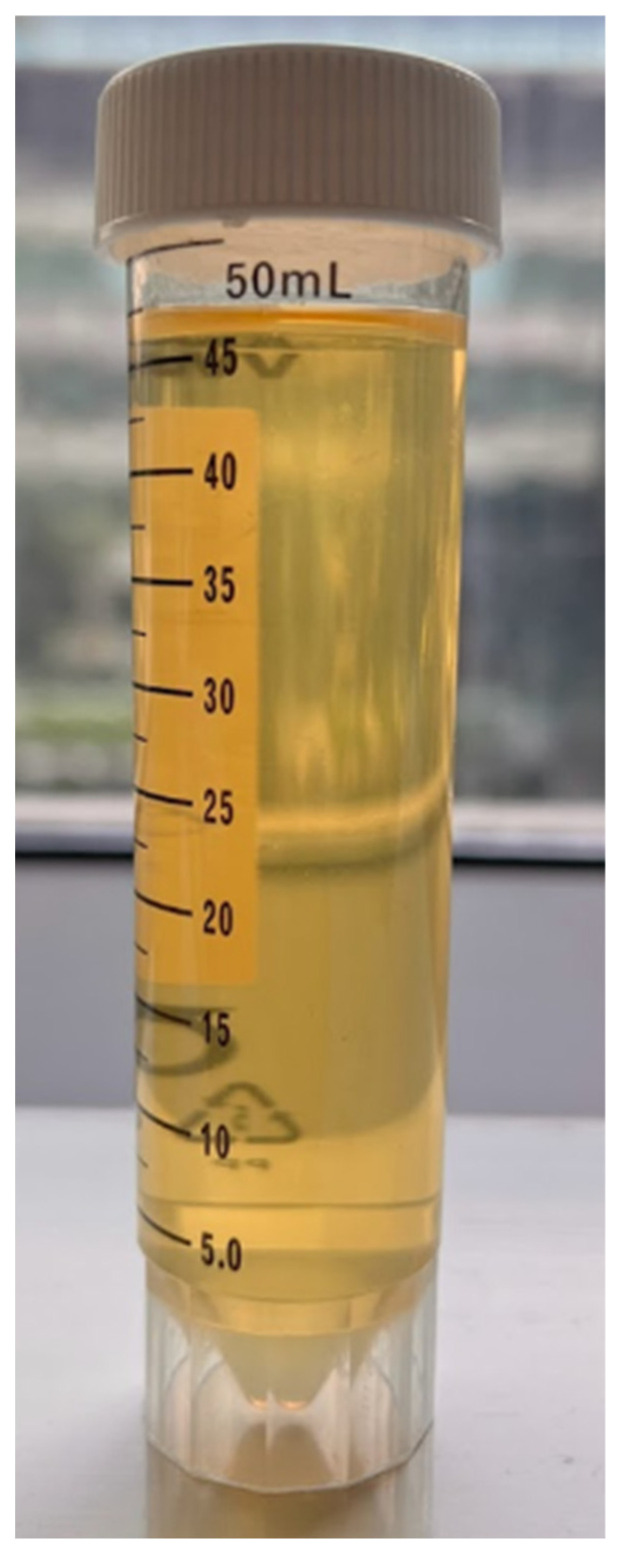
Visual appearance of the fluid after the breaker test using sodium bromate (5 ppt) at 300 °F. Effluent is clear, with no visible polymer residue, indicating effective gel breaking.

The effluent of the viscometer run was collected after the test and visually inspected. It showed a visually clear sample without visible polymer residue, as seen in Figure 14. This is due to using a guar derivative with minimal impurities (CMHPG). However, further tests such as fracture conductivity and coreflooding must be completed to properly assess the tendency to cause formation damage.

### 2.4. Proppant Suspension

**Figure 15:**
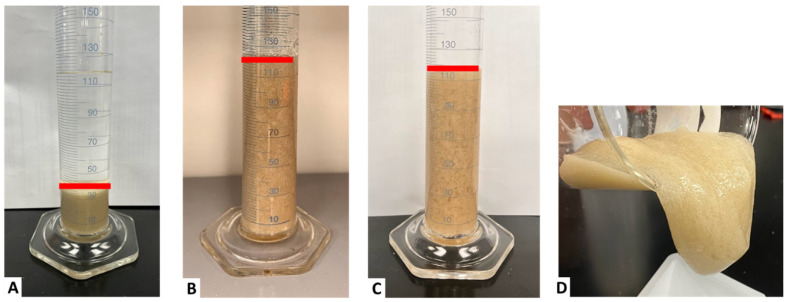
Proppant suspension performance of CMHPG fluid with 4 ppg of 40/70 mesh proppant after 24 h: (**A**) hydrated CMHPG only, room temperature, showing full settling of the added proppant. (**B**) Crosslinked with 4 gpt of Zr-lactate + 2 ppt of sugar alcohol, room temperature showing >95% proppant suspended. (**C**) Same formulation aged at 200 °F showing >95% proppant suspended. (**D**) Lip formation after heating showing stable gel structure retained and >95% proppant suspended. The red line indicates the proppant suspension level in the treatment fluid.

The results are seen in Figure 15, showing that the hydrated fluid without the crosslinker was unable to suspend the proppant (Case A). The fluid in the presence of the sugar alcohol and the crosslink was able to suspend the proppant at room temperature conditions for 24 h (Case B). The last case shows that the fluid in the presence of the sugar alcohol and the crosslink could hold the proppant at 200 °F for 24 h (Case C). The last photo (Case D) shows the lip formation after heating the fluid and its ability to hold >95% of the proppant.

## 3. Summary and Conclusions

This study demonstrates the effectiveness of sugar alcohols as crosslinking delay additives for high-temperature fracturing fluids. Laboratory testing was performed using a CMHPG-based fluid system crosslinked with zirconium lactate at a high pH. The evaluation included rheological performance, proppant suspension, and compatibility with oxidizer-based breakers.

Key findings are summarized below:

1. CMHPG fluids crosslinked at a pH of 10.7 exhibited rapid and unstable crosslinking behavior across 1–4 gpt of zirconium concentrations at 300 °F, leading to viscosity loss under shearing.
2. Incorporating sugar alcohol successfully delayed crosslinking, with optimal results observed at 2 ppt of sugar alcohol combined with 4 gpt of zirconium crosslinker.
3. The delayed crosslinking effect improved viscosity stability, enabling fluids to maintain viscosity above 300 cP for 1.5 h at 300 °F.
4. Breaker tests using sodium bromate confirmed that sugar alcohols do not interfere with oxidative breakers, providing a controlled viscosity reduction profile.
5. Proppant suspension was retained by over 95% of the proppant under both room temperature and 200 °F, demonstrating the additive’s suitability for field applications.

These results highlight the potential of sugar alcohols as a safer and effective alternative to conventional delay additives in fracturing fluids. Further evaluation in dynamic flow test and coreflood experiments, especially with higher salinity water conditions, will be pursued in future studies to assess long-term formation compatibility and cleanup performance.

## 4. Recommendations

It is recommended to use at least 40 lb/1000 gal of CMHPG at a pH of 10 for high-temperature conditions of 300 °F. The tests also recommend using 4 gpt of Zr-Lactate crosslinked with a concentration of 2 ppt of sugar alcohol. It is highly advised to test the fracturing fluid with the field mixing water, as salinity can play a huge role in the additive interactions. When utilizing other ligands of zirconium crosslinkers, tests should be pursued to find the optimal ratio between the sugar alcohol additive and the crosslinker used.

## 5. Materials and Fluid Preparation

Carboxymethylhydroxypropyl guar (CMHPG-powder), Tetraethylenepentamine (TEPA-liquid at 50 wt.%), and a sodium bromate live breaker were acquired from a service company. Zirconium lactate was obtained from a chemical company with a ZrO_2_ concentration of 5.5–5.7 wt.%. The 40/70 mesh proppant used for the proppant suspension tests was received from an ongoing field treatment. All these commercial additives were used as received. The sugar alcohol is composed of six carbon atoms and multiple hydroxyl groups, commonly used in consumer products, recognized for its safety. It is a commercially available, food-grade polyol with six carbon atoms and multiple hydroxyl groups in powder form at a purity of >99 wt.%. Tap water (<500 ppm) was used to prepare the fracturing fluids, as it closely resembles the water quality in the targeted field trial. The fracturing fluid was prepared fresh before testing to eliminate any degradation/aging influence. No biocides, surfactants, oxygen scavengers, or external gel stabilizers were used in these tests to evaluate the influence of the sugar alcohol additive on the rheological response of the fracturing fluids.

To prepare the 40 lb/1000 gal (40 ppt) of fracturing fluid, 3.84 g of CMHPG powder was added to 800 mL of tap water and blended at 800+ rotations per minute (RPM) for 15 min to reach the full hydration status of the polymer. The blending speed is determined based on the vortex formation. A strong vortex without bubble formation is preferred to accelerate hydration.

A sample of the fluid was regularly taken to monitor the consistency of the hydrated gel. At room temperature, the hydrated gel achieved a viscosity of ~34 cp at 300 RPM. In rare cases where the fluid did not reach that viscosity, it was returned to the blender and mixed for 10 more minutes. Following the polymer hydration quality check, the fluid was transferred to a 1 L beaker and left still for 1 h to reduce the number of bubbles present in the solution.

A volume of 250 mL of the hydrated 40 lb/1000 gal fluid was then measured using a graduated cylinder, transferred to the blender, and mixed at a lower RPM (<400) to avoid the formation of additional air bubbles. Reducing the amount of air bubbles at this point is critical because once the fluid is crosslinked, the removal of the bubbles becomes difficult due to the high viscosity. Additionally, air bubbles are undesired as they could produce oxygen radicals at higher temperatures, which can break the polymer chains [5]. This preparation process was followed carefully to maintain consistency in the tests. After that, TEPA was added at a concentration of 1 gallon/1000 gal (gpt) (0.25 mL in the 250 mL) to raise the pH to a value between 10 and 11. The sugar alcohol was then measured to the desired concentration and dissolved in 1–2 mL of tap water to ease the addition to the hydrated fluid in the blender. At this stage, live breakers were added, as necessary, in a similar manner to the sugar alcohol. Finally, the crosslinker was added at the desired concentration. The fluid was then allowed to mix for 10 s to incorporate all the additives evenly within it. The vortex was monitored for a duration of 30 s, and the closure time was recorded if it occurred within this time limit. After preparing the fracturing fluid, 50–52 mL of this fluid was measured and transferred to the HPHT rheometer cup.

## 6. Equipment and Laboratory Procedures

### 6.1. HPHT Viscometer

A high-pressure/high-temperature rotational viscometer with a bob and cup setup is used for measuring viscosity at 300 °F. The rheometer requires a fluid sample between 48 and 54 mL. The instrument is equipped with a carbon-block electric heater rated up to 500 °F, and the equipment is rated up to 2000 psi.

The test schedule used follows the ISO 13503-1 standard procedure for viscosity measurements [54]. This schedule consists of measuring viscosity at a shear rate of 100 s^−1^ for most of the tests, with short variable shear rate ramps in between. The low shear ramps generate the viscosity artifacts seen in the figures. The change in shearing rate is favorable to determine the performance of the fracturing fluid at the various stages of the hydraulic fracturing treatment. Higher shear rate values can be correlated to the performance in the tubular, while lower shear rates would depict the behavior inside the fracture. The performance before and after the shear ramps gives us an indication of how stable the crosslinking bonds are. An anti-climber is used to prevent cases where high-viscosity fluid would climb up the bob and shaft, causing errors in the readings.

After installing the anti-climber and bob, the machine is “tared” to reset the viscosity readings to zero. The cup containing the fracturing fluid sample is then attached and tightened. Pressure is added to the cup using nitrogen (~500 psi) to prevent the boiling of the sample during the test. Simultaneously, to compare the additive influence properly, the heater is manually preheated to 150 °F, which ensures a consistent starting profile for the variable concentrations of the additive. Once the heater achieves the desired preheating temperature, it is raised to contact the cup, and recording is initiated. The instrument records viscosity measurements every 5 s for a duration of 90 min. The starting conditions are particularly important for this work due to the sugar alcohol’s potential ability to delay crosslinking. It is of interest to study how high the initial viscosity is and how fast the buildup of the crosslinking viscosity is. Although viscosity is not a direct prediction of the ability to carry proppant, a crosslinked fluid viscosity of at least 100 cP can successfully prevent failure during the pumping operation.

### 6.2. Lipping Behavior

As soon as the fluid is placed in the rheometer for testing, the remaining sample is collected in a plastic bottle to check for its lipping capability at room temperature. The bottle is tilted in such a way that the fluid pours into a glass beaker. This visually simulates the fluid’s lipping behavior during surface-to-reservoir pumping. Knowing the visual behavior of the fluid can help us understand the integrity of the fluid and the likelihood of screening out. The results should also help in confirming the viscosity results. The lower the viscosity, the more water-like the fluid flow is. It also serves to identify if the fluid exhibits over-crosslinking properties, if it can easily be sheared, and its re-healing capability after shearing.

### 6.3. Proppant Settling

Proppant settling tests were conducted at atmospheric pressure and 200 °F using borosilicate glass graduated cylinders and a convection oven. A proppant concentration of 4 lb/1000 gal (ppg) of 40/70 mesh (~48 g for each 100 mL) was used at the desired condition. The proppant was added to the fracturing fluid in a 500 mL beaker and mixed thoroughly to incorporate the proppant within the fluid. Following that, each fluid was placed in the graduated cylinder. Each test was aged for 24 h, and photographs were taken to compare the proppant suspension ability across different cases.

Three cases were made to assess the ability to suspend proppant. The first case was a non-crosslinked (hydrated) fluid. The second case contained the sugar alcohol and the crosslinker prior to initiating crosslinking (prior to heat exposure). The third case contained the sugar alcohol and crosslinker after initiating crosslinking at 200 °F.

## Data Availability

The original contributions presented in this study are included in the article. Further inquiries can be directed to the corresponding author(s).

## Abbreviations

The following abbreviations are used in this manuscript:

HPG	Hydroxypropyl Guar
CMHPG	Carboxymethylhydroxypropyl Guar
cP	Centipoise
gpt	Gallons of chemical per thousand gallons of treatment
HPHT	High-pressure/high-temperature
lb	Pounds
ppg	Pounds of chemical per gallon of treatment
ppt	Pounds of chemical per thousand gallons of treatment
TEPA	Tetraethylenepentamine
